# Merging single-shot XFEL diffraction data from inorganic nanoparticles: a new approach to size and orientation determination

**DOI:** 10.1107/S2052252517012398

**Published:** 2017-09-22

**Authors:** Xuanxuan Li, John C. H. Spence, Brenda G. Hogue, Haiguang Liu

**Affiliations:** a Complex Systems Division, Beijing Computational Science Research Center, 8 East Xibeiwang Road, Haidian, Beijing 100193, People’s Republic of China; b Department of Engineering Physics, Tsinghua University, 30 ShuangQing Rd, Haidian, Beijing 100084, People’s Republic of China; c Department of Physics, Arizona State University, Box 871504, Tempe, AZ 85287, USA; d Biodesign Institute, Biodesign Center for Immunotherapy, Vaccines and Virotherapy, Biodesign Center for Applied Structural Discovery, School of Life Sciences, Arizona State University, Tempe, AZ 85287, USA

**Keywords:** orientation determination, structure heterogeneity, single-particle scattering, nanoparticles, core–shell architecture, XFELs

## Abstract

X-ray free-electron lasers (XFELs) provide new opportunities for structure determination of biomolecules, viruses and nanomaterials. With unprecedented peak brilliance and ultra-short pulse duration, XFELs can tolerate higher X-ray doses by exploiting the femtosecond-scale exposure time, and can thus go beyond the resolution limits achieved with conventional X-ray diffraction imaging techniques. Using XFELs, it is possible to collect scattering information from single particles at high resolution, however particle heterogeneity and unknown orientations complicate data merging in three-dimensional space. Using the Linac Coherent Light Source (LCLS), synthetic inorganic nanocrystals with a core–shell architecture were used as a model system for proof-of-principle coherent diffractive single-particle imaging experiments. To deal with the heterogeneity of the core–shell particles, new computational methods have been developed to extract the particle size and orientation from the scattering data to assist data merging. The size distribution agrees with that obtained by electron microscopy and the merged data support a model with a core–shell architecture.

## Abstract 2

An algorithm is presented to recover the orientations of nanoparticles with size variations from X-ray free-electron laser (XFEL) scattering.

## 1. Introduction   

The realization of the ‘diffraction-before-destruction’ experimental approach at X-ray free electron laser (XFEL) facilities, such as the Linac Coherent Light Source (LCLS) at SLAC National Laboratory (Menlo Park, California, USA), makes it possible to outrun radiation damage (Chapman *et al.*, 2006 ▸) using ultrashort X-ray pulses (Emma *et al.*, 2010 ▸). As fully coherent X-rays, XFEL pulses are unprecedentedly bright, promising scattering measurements at high resolution from noncrystalline samples such as macromolecular complexes or viruses [for a review of the LCLS design and applications, see Bostedt *et al.* (2016 ▸)]. Coherent diffraction imaging (CDI) of single particles (with one particle per shot) will eventually enable the determination of biomolecular structures without the need to grow crystals (Miao *et al.*, 2015 ▸; Gallagher-Jones *et al.*, 2016 ▸). The reconstruction of a three-dimensional real-space density map based on a set of scattering patterns from many randomly oriented and structurally similar particles remains a field of active research, including the development of algorithms for accurate determination of particle orientation to allow the merging of data sets into a three-dimensional diffraction volume, followed by solution of the phase problem and reconstruction to a real-space electron-density map.

From the perspective of data analysis, the orientation determination and the treatment of sample heterogeneity are two challenging problems for XFEL single-particle imaging. It is understood from recent cryo-electron microscopy (cryo-EM) studies that the ability of algorithms to distinguish small changes in particle orientation from small changes in structure (from projection data) is crucial for the formation of mol­ecular movies from an ensemble, and is hence a problem of the greatest importance in structural biology. Heterogeneity of synthetic nanoparticles arises mainly from variations in particle size. In the case of virus particle scattering studies, even for identical icosahedral virus particles, whose structure is defined by the outer capsid protein and the inner DNA/RNA molecules, the lack of apparent homogeneity most likely arises from variations in the thickness of the hydration shell, or from precipitates condensing on the virus surface, or from variations in the packing of the genome. The orientation recovery must be done in the presence of beam shot-noise and instrument limitations, such as background scattering from apertures, shot-to-shot intensity variations, detector read-out noise, nonlinear detector response and other unidentified noise sources. Several algorithms have been proposed for orientation determination for single-particle scattering data. Common arc (or common line) methods (Shneerson *et al.*, 2008 ▸; Bortel & Tegze, 2011 ▸) rely on finding the intersecting lines between scattering patterns, due to the fact that all patterns must pass through the origin of reciprocal space. The expansion, maximization and compression (EMC) algorithm simultaneously improves an estimate of orientations and the merged intensity (Loh & Elser, 2009 ▸). The manifold embedding method maps each scattering pattern onto the SO(3) rotation space, based on the assumption that similar patterns, represented by a vector in higher dimensions, lie close together, and their orientation can be ordered because they must fall on a path which is a closed loop for a full rotation (Ourmazd *et al.*, 2010 ▸; Hosseinizadeh *et al.*, 2014 ▸). This method has recently been used to obtain the first experimental conformational movie of an icosahedral virus and determination of its reaction coordinate during extrusion of a viral genome (Hosseinizadeh *et al.*, 2017 ▸). The EMC algorithm has been applied to recover orientations from experimental data, leading to a three-dimensional reconstruction of the mimivirus data collected at LCLS (Ekeberg *et al.*, 2015 ▸). Some of these algorithms can be further adapted to address the problems of sample heterogeneity. There are successful cases demonstrating the application of these algorithms in handling heterogeneous sample data, where Kassemeyer *et al.* (2013 ▸) used the geodesic and in-plane rotations algorithm (GIPRAL) to select the CDI data that correspond to the same sized particles. Detailed discussions of these approaches were recently reviewed (Liu & Spence, 2016 ▸). In the related field of single-particle cryo-EM, where real-space images solve the phase problem and the Friedel symmetry is not imposed, the maximum likelihood method with Bayesian statistics (Scheres, 2012 ▸) has been used to iteratively classify images with different conformations by sorting them according to both orientation and a limited number of conformational classes. The manifold embedding method has also been used to demonstrate its potential for mapping cryo-EM images into conformational space (Dashti *et al.*, 2014 ▸). These approaches can possibly be applied to classify and merge XFEL single-particle scattering data from samples of unknown orientation in the presence of sample heterogeneity.

Synthetic nanoparticles have been used in X-ray coherent imaging research for proof-of-principle experiments. The reconstructions of nanoparticles have been successfully produced from coherent X-ray imaging data, using either Bragg scattering for small crystals or low-angle scattering for single particles (Robinson *et al.*, 2001 ▸; Williams *et al.*, 2003 ▸; Kassemeyer *et al.*, 2013 ▸). The full potential of the LCLS for single-particle imaging is still under investigation (Aquila *et al.*, 2015 ▸). In the work described here, inorganic core–shell nanocrystals consisting of a palladium (Pd) outer shell and a gold (Au) core were used as a surrogate model for single virus particle experiments at LCLS. These inorganic particles, with their sharp crystallographic external facets (reminiscent of the facets of icosahedral viruses) and a different internal symmetry, provide an ideal test sample for developing methods that can later be generalized for application to virus structural biology. Detailed descriptions of the sample and the data have been reported previously, along with preliminary analysis results (Li *et al.*, 2017 ▸). In that work, a subset of the experimental data composed of the 32 scattering patterns from particles that had one flat face normal to the incident X-ray direction was analyzed, and the particle size information was found to be consistent with scanning tunneling electron microscopy (STEM) results.

The general case with arbitrary orientation relative to the incident beam is described here with a new method that utilizes prior information on the particles to develop a reference-based analysis approach. To tackle the particle size heterogeneity challenge, an angular intensity profile was defined to improve the model comparison accuracy by reducing the influence of particle size variations. As a result, the orientations of 10 878 out of 54 405 scattering patterns [20%, compared with 0.4% in Li *et al.* (2017 ▸)] were recovered at a reasonable confidence level. The size distribution obtained from the X-ray data is consistent with the results from electron microscope imaging. The orientation distribution indicates a bias towards the orientations that produce stronger scattering features. The majority of the scattering patterns and merged data suggest that a model with a core–shell heterogeneous structure is favoured over other alternatives.

## 2. Methods   

### 2.1. Samples   

**Figure 1:**
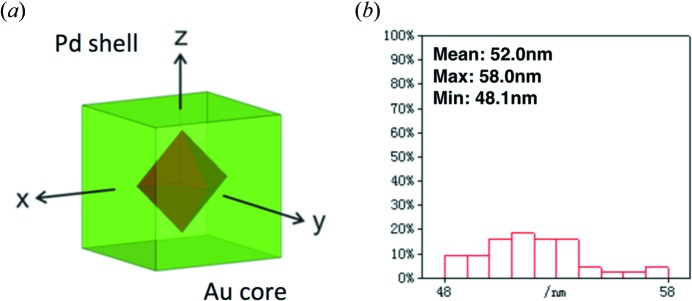
Information for the core–shell nanoparticle. (*a*) Schematic drawing of an Au–Pd core–shell nanoparticle. The green cube indicates the outer shell of the particle composed of palladium (atomic number 46), while the red core is composed of gold (atomic number 79). (*b*) Size distribution of nanoparticles obtained from electron microscope imaging. The sizes range from 48.1 to 58.0 nm, with a mean value of 52.0 nm.

Au–Pd core–shell nanocrystals were prepared at the National Tsinghua University using procedures described previously (Yang *et al.*, 2011 ▸; Li *et al.*, 2017 ▸). The synthetic particles exhibit a cube-shaped palladium shell and a regular octahedral gold core, with a mean size (length of the cube edge) of 52 nm (Fig. 1 ▸).

### 2.2. Experimental setup   

Experimental details are as described by Li *et al.* (2017 ▸). Briefly, the nanoparticles were suspended in water and delivered to the XFEL beam on the Coherent X-ray Imaging (CXI) beamline at the LCLS using the nanofocus chamber (Liang *et al.*, 2015 ▸). The X-ray energy was set to 6.0 keV (λ = 2.06 Å). The XFEL pulse duration was ∼60 fs with a repetition rate of 120 Hz. The gas dynamic virtual nozzle (GDVN) system was used for injection (Weierstall *et al.*, 2012 ▸). Settling of the sample in the syringe during injection was prevented by using a slowly rotating temperature-controlled syringe holder (Lomb *et al.*, 2012 ▸). The Cornell–SLAC Pixel Array Detector (CSPAD) was positioned 565 mm from the sample in the far field. The CSPAD panels were arranged in a geometry effectively covering an area of 1748 × 1748 pixels, with a pixel size of 110 × 110 µm. However, the actual useful region of the detector was effectively reduced to an annulus bounded by two circles with radii of 75 and 150 pixels. The signal within 75 pixels could not be measured due to the hole in the CSPAD arrangement, while the signal beyond 150 pixels is weak and mixed with background scattering intensity. The region of interest (the annulus) corresponds to momentum transfer vectors of modulus *q* within the range 0.44 ≤ *q* ≤ 0.89 nm^−1^ (resolution *d* = 2π/*q* = 14.27 to 7.06 nm). The Ewald sphere is approximately flat in this resolution range, so Friedel’s law is applicable to the two-dimensional scattering patterns. The *Cheetah* program was used for hit-finding (Barty *et al.*, 2014 ▸).

### 2.3. Data analysis   

Mapping the scattering intensity recorded on a two-dimensional detector into a three-dimensional diffraction volume is a critical step towards determining the three-dimensional electron density of objects in real space. Using the GDVN injection system, the sample orientations cannot be controlled or measured directly. Therefore, the orientation information must be recovered from the diffraction patterns using computational algorithms. Heterogeneity of samples, including size variation or conformational changes, increases the difficulty in determining particle orientations. In the analysis of this data set, prior information about the sample particles was utilized to disentangle the unknown orientations and sample heterogeneity, with the assumption that size variation is the dominant component causing heterogeneity.

#### 2.3.1. Orientation determination   

The nanoparticles exhibit differences in size, composition of the core and outer shell, and certain defects at the surface of the particles, but for our analysis we assumed that the overall size variation is the most pronounced effect. Based on images obtained from electron microscopy, we first constructed a reference model with a cubic palladium shell and an octahedral gold core (Fig. 1 ▸). The corresponding Fourier transform was computed from this reference model to simulate the scattering intensity function in reciprocal space. Here, we focused mainly on the results obtained using this reference model to investigate the orientation distribution and size variation of the nanoparticles that were intercepted by XFEL pulses. Given the computed intensities in the three-dimensional diffraction volume, two-dimensional slices corresponding to the Ewald surface cutting through reciprocal space were extracted to simulate patterns with orientations that sampled the SO(3) space. Each experimental pattern was then compared with the simulated reference patterns and a similarity score was assigned using the Pearson correlation coefficient (denoted Pcc hereafter). Considering that the intensity correlation at the pixel level is very sensitive to variations in particle size, each two-dimensional scattering pattern was converted to a one-dimensional angular intensity profile, which was used to calculate the Pcc between the experimental data and the reference patterns. The most probable orientation can then be identified by locating the reference pattern with the highest Pcc value to the corresponding experimental pattern.

**Figure 2:**
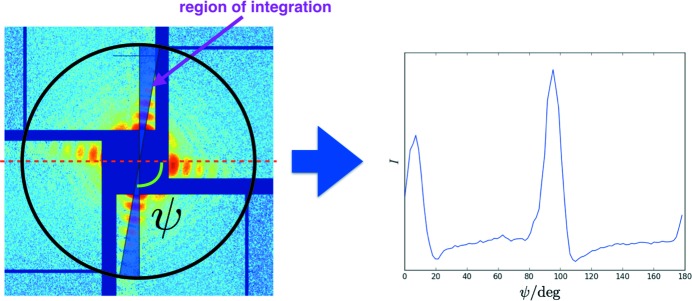
Angular profile conversion. The average intensity profile as a function of azimuth angle (right) is extracted from the raw pattern (left) (two sectors are plotted to show the region of integration at angle ψ).

Each two-dimensional pattern was divided into 100 equally spaced sectors (pie slice, see Fig. 2 ▸) around the scattering center (*i.e.* the origin of Fourier space). The mean value of the intensities was then calculated within each sector for valid pixels (defined according to the experimental detector setup), 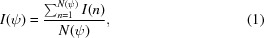
where *N*(ψ) is the number of valid measurements (after masking out dead pixels, water streak scattering and detector gaps) in the sectors defined by [ψ, ψ + Δψ] and [180 + ψ, 180 + ψ + Δψ] (due to Friedel symmetry); here Δψ = 1.8°. *I*(*n*) is the *n*th intensity value. *I*(ψ) is the average intensity in the two sectors. The same mask that removes invalid pixels in the experimental data was applied to each reference pattern before the profile calculation. As a result, each pattern was converted to an average intensity profile as a function of the azimuth angle ψ (Fig. 2 ▸).

The Pearson correlation coefficient Pcc between the experimental and reference profiles is therefore calculated as: 
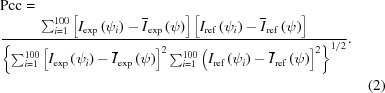
Although averaging the intensity during the angular profile conversion results in information reduction compared with the raw data, this approach is robust against the size variation of the particles and computationally efficient. To balance the contributions of the high- and low-intensity values to the correlation calculation, different weighting schemes were tried. It was found that the logarithmic value of the averaged intensities yielded optimal results. In the following analysis, logarithmic values of the one-dimensional averaged intensity from each pattern were used for comparison, to identify the most probable orientation of the sample for each experimental pattern.

The normalized angular intensity profile described in the previous paragraph is size-invariant if the momentum transfer |*q*| is integrated out, as shown in Appendix *A* [App appa]. In the experimental analysis, the integration range for |*q*| is limited from *q*_min_ to *q*_max_ (here, 0.44 ≤ *q* ≤ 0.89 nm^−1^), making the integration not fully size-invariant. However, the results showed that the orientation recovery method is still robust against variations in particle size because this angular profile is more specific to the particle orientation than to the particle size (see *Discussion* section[Sec sec4]).

#### 2.3.2. Size determination   

Sorting the size information from the scattering patterns is necessary before assembling the scattering intensity into a three-dimensional volume. The particle size can be derived from the *q* spacing between the interference fringes (speckles) in the scattering patterns, as described for X-ray scattering from a faceted nanocrystal by equation (1) of the paper by Kirian *et al.* (2010 ▸), with Δ*k*_*Z*_ = 0 (for the projection approximation with a flat Ewald sphere) for Bragg reflection **g** = 0. These are exactly analogous to the *N* − 1 subsidiary maximum observed between Bragg reflections from an optical grating of *N* slits. The *q* spacing running along the diffraction streaks also depends on the orientation, which can be recovered using the approach described in the previous section. A computer program was implemented to identify the spacing between speckles. It consists of three steps: (i) calculate the angular intensity profile to find the angle θ_max_ associated with the maximum intensity (*i.e.* identifying the streaks); (ii) find the speckles along the line at θ_max_; (iii) calculate the average spacing between adjacent speckles. This *q* spacing, projected onto the detector and denoted Δ*x*, was then used to derive the particle sizes.

According to the geometric relationship between cubic facets and the X-ray incident direction, the patterns were classified into two cases: (i) the normal incidence case and (ii) the general incidence case. For the normal incidence cases, the incident beam is perpendicular to one of the six cubic faces, and the resulting scattering patterns exhibit two pronounced series of speckles that cross perpendicularly. In this case, the particle size, *d*, can be estimated directly from the spacing between speckle peaks at low resolution, as reported by Takahashi *et al.* (2013 ▸). Given the experimental setup, the particle size is obtained as 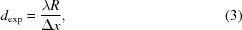
where λ is the X-ray wavelength, *R* is the distance between the sample and the detector, and Δ*x* is the spacing between adjacent speckles on the detector.

For the general incidence cases, the X-ray beam is not normal to any face of the cubic shell. In such cases, it is much more challenging to compute the particle sizes directly based on the gap between the fringes, so we resort to the reference-based approach by comparing the experimental patterns and the simulated patterns at the *same* orientation. For those patterns with clear streaks, the spacing between speckles can be used to derive particle sizes by utilizing the inverse relation between real space and Fourier space 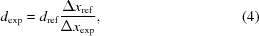
where *d*_exp_ and *d*_ref_ are the particle sizes used in the experiment and the simulation, respectively, and Δ*x*_exp_ and Δ*x*_ref_ are the spacings between speckles in the experimental and simulated patterns, respectively. In principle, the particle size for the general incidence cases can also be calculated by measuring a series of *q* spacings and solving the resulting linear equations. In this experimental data set, the *q*-spacing variation along the detected streaks is very small (<1% of the detector pixel size), so it is not practical to obtain particle size information directly from the data.

## 3. Results and analysis   

### 3.1. Method validation using simulation data   

#### 3.1.1. Orientation determination: at the pixel level *versus* integrated angular profile level   

We assessed the performance of two comparison approaches, namely, pixel-wise comparison and angular profile comparison. The raw data are in the form of two-dimensional scattering patterns consisting of *n* pixels, so it is straightforward to conduct pixel-level correlation calculations by treating each pattern as a vector in an *n*-dimensional space. If there is no size variation, this approach is valid and accurate. However, the two-dimensional patterns are strongly dependent on particle size, according to the scaling properties of Fourier transforms. More specifically, the intensity distribution in the radial direction has strong oscillations for scattering patterns resulting from objects with facets, observed as speckles in the scattering patterns (see Fig. 2 ▸, left). The positions and values of the minima and maxima are determined by the particle size and orientation. On the other hand, the angular profile approach described in the *Methods* section[Sec sec2] allows integration of the intensity along the radial direction, *i.e.* the modulus of momentum transfer, *q*. As a result, the size information is partially integrated out (see Appendix *A* [App appa]), making the angular profile depend strongly on orientations and be less sensitive to particle size variation. Although the actual *q* range for integration is not ideally from 0 to infinity in real experimental data analysis, it is shown that the angular profile is still far less sensitive to particle size variations compared with the two-dimensional patterns.

The method was validated using scattering data simulated for particles sized between 44 and 60 nm with an increment of 2 nm. A 52 nm model, close to the mean size of the nanoparticles used in the experiment, was constructed using *a priori* core–shell model information as the reference. The Euler angle space of rotation was confined to a subspace, where 70° < α, β, γ < 90°, and the step angles Δα, Δβ, Δγ were set to 2°. This choice of subspace is possible because of the high symmetry of the nanoparticles, so that the recovered orientations do not have degeneracies, *i.e.* the core–shell particle will not resemble itself using the rotations within this subspace. To be consistent with the experimental data, the scattering intensity within two annuli (0.44 and 0.89 nm^−1^) was used to simulate the experimental conditions. The particle size information was only used for validation purposes.

**Figure 3:**
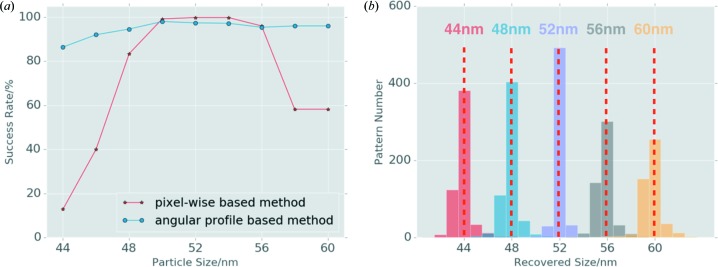
Orientation recovery and size analysis for simulated data. (*a*) Orientation recovery results with the pixel-level method and the angular profile method. The angular profile method (blue curve) is more robust in the presence of size variation than the pixel-level method (red curve). (*b*) Size determination for the simulated data. The red dashed lines indicate the actual values of particle size, while the histograms show the size distribution obtained using our method. The particle sizes are narrowly distributed, and the differences between the mean values of each group and the actual values are within 0.5%.

The recovery correctness criterion was 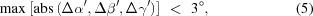
where 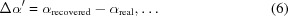
The orientation recovery results for both pixel-level based and angular-profile based algorithms are summarized in Fig. 3 ▸(*a*). Using a pixel level comparison, more than 90% of the simulated patterns are recovered to their correct orientations when the particle sizes are within 2 nm of the reference model. The accuracy drops rapidly when the particle size deviates from the reference model size. The angular-profile based approach is more robust in determining the correct orientations, even for large size differences (up to ±8 nm). From Fig. 3 ▸(*a*), we can conclude that the pixel-level method performs slightly better only when the experimental sample size distribution is narrow and known. For nanoparticle scattering data, the angular-profile based comparison approach is more appropriate.

#### 3.1.2. Size determination   

To validate the size determination method, 5000 scattering patterns were simulated for particles with sizes covering the same range as the orientation recovery test described in the previous section (44, 48, 52, 56 and 60 nm, 1000 patterns at random orientations for each size). A 52 nm core–shell particle was used as the reference to determine the particle sizes. Using the algorithm described in the *Methods* section[Sec sec2], the results indicate that, for all data sets, the recovered particle sizes are narrowly distributed around the actual values. The difference between the average size for each group and the true size is within 0.5% (Fig. 3 ▸ *b*).

### 3.2. Analysis results for the XFEL single-particle scattering data   

From the raw data collected at LCLS, 54 405 experimental patterns were identified to be scattering patterns from the Au–Pd core–shell nanoparticles, as reported previously (Li *et al.*, 2017 ▸). Because of the symmetry of the reference model, the orientations can be covered using a subspace of the SO(3) group, and here we confine the Euler angles to be from 0 to 90°. The discretization size was set to 1° for the α, β and γ angles, resulting in 753 571 (91^3^) reference profiles to be compared with the experimental data.

#### 3.2.1. Orientation recovery   

**Figure 4:**
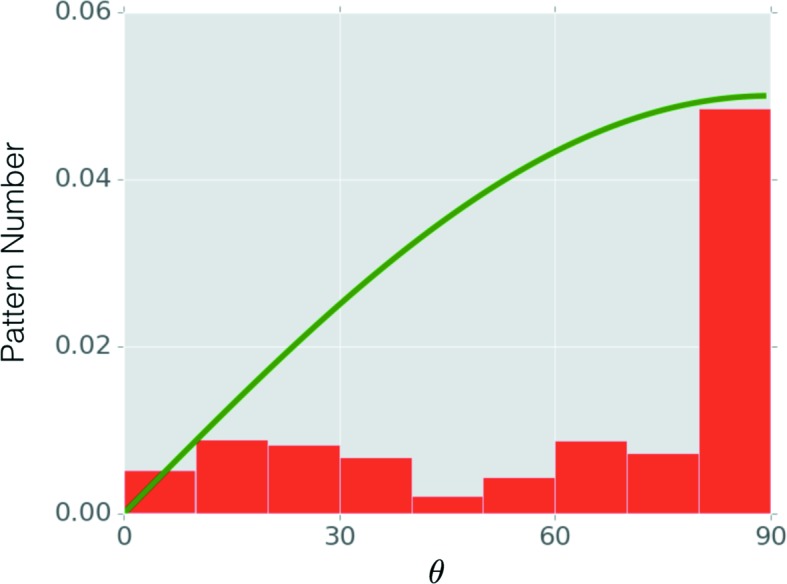
Tilting-angle distribution for 10 878 experimental patterns. The angle θ is the tilting angle, defined as the angle between the incident beam direction and the direction that is normal to the cubic face, such as the *z* axis of the particle (see Fig. 1 ▸ *a*). The green curve is the expected distribution, assuming random orientations. The green curve illustrates that the analytical distribution function *p*(θ) is proportional to sin(θ).

The orientations of the nanoparticles corresponding to scattering patterns in the experiment are not known in advance, so it is difficult to assess the performance of orientation recovery directly. Instead, we studied the orientation distribution by grouping the recovered orientations based on the second Euler angle, β. This angle can be pictured as the angle between the direction pointing from a cubic face towards the particle center and the incident beam direction. For example, if the incident beam direction coincides with the −*z* direction for the reference model before rotation, then the rotation of the particle will change the angle between the beam direction and the −*z* direction by β. To differentiate this from the Euler angles, we denote this angle as the tilting angle, θ. The analysis was done on 10 878 patterns that were selected automatically from the overall set of 54 405 patterns. The angle θ exhibits the distribution shown in Fig. 4 ▸. This is different from the expected distribution for randomly oriented particles, which should be an increasing function (proportional to sinθ, which can be derived by uniformly sampling points on a spherical surface) as indicated by the green curve.

The deviation of the observed distributions from the expected distribution could be attributed to two possible sources: (i) the orientation preference of the sample particles, probably related to flow alignment in the liquid jet stream used to deliver the particles across the pulsed XFEL beam; or (ii) a deficiency in the data analysis programs.

**Figure 5:**
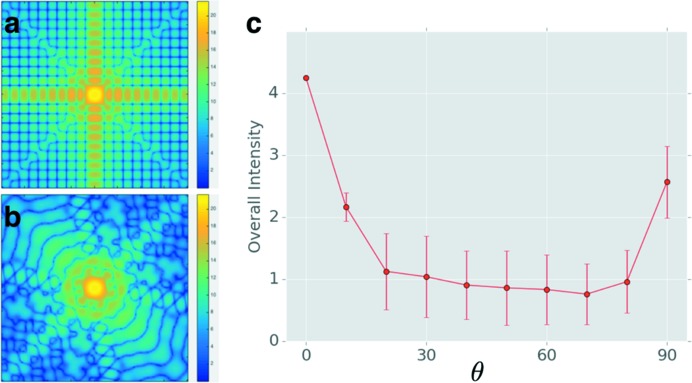
The dependence of the scattering pattern on orientation. (*a*) A simulated pattern for a tilting angle of 0°, the normal incidence case. (*b*) A typical scattering pattern when the tilting angle is 45°. There are two strong streaks when the incident beam is perpendicular to the particle surface as in panel (a), but the features for the scattering pattern at a tilting angle of 45° are less pronounced. (*c*) Total intensity plotted as a function of tilting angle. We simulated 500 patterns for each tilting angle. The overall intensity within the *q* range 0.44–0.89 nm^−1^ is calculated, showing that the overall intensity is lower for cases where the tilting angle is around 45°, while the standard deviations (error bars) for the intensity indicate larger fluctuations for the same group of patterns.

We simulated patterns at different tilting angles and observed that scattering patterns with medium tilting angles (around 45°) do not exhibit strong speckle features and that the overall measurable scattering intensity (averaged from 500 patterns simulated at each tilting angle) was much lower (Fig. 5 ▸). After systematic analysis, we found that the under-represented orientations with tilting angles around 45° are more likely to trace back to the orientation recovery algorithm, which depends on strong features that reflect the orientations. In other words, the core–shell model does not yield distinct scattering features when the tilting angle is around 45°, leading to a failure to identify the patterns at those orientations. Furthermore, due to the presence of experimental noise, the patterns that produced less pronounced features yielded low correlation coefficients compared with the reference profiles and were classified as failed cases in the orientation recovery analysis, because the correlation coefficient is below the pre-set threshold level. If we reduce the confidence levels by lowering the correlation coefficient threshold, the number of patterns around 45° increases (see Fig. S1 in the supporting information). Therefore, the proposed algorithm works well for patterns with good features, but fails for featureless patterns if we set a high threshold to ensure the accuracy of the recovered orientations.

#### 3.2.2. Particle size analysis   

**Figure 6:**
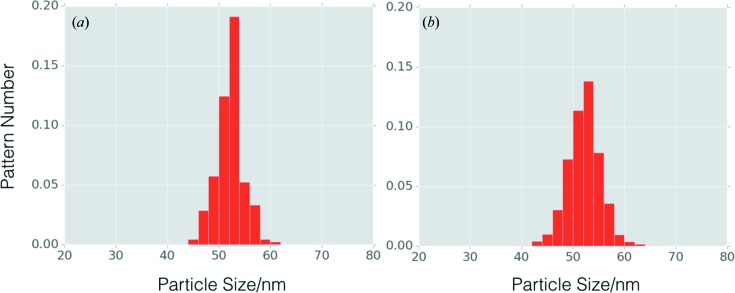
Particle size distribution. (*a*) Size distribution of particles in normal incidence cases. Note that the distribution is slightly different from the plot given by Li *et al.* (2017 ▸), due to the different number of patterns in the analysis. (*b*) Distribution of particle sizes in the general incidence case. The two distributions both show that the mean size of particle is approximately 52 nm, consistent with electron microscopy image statistics.

Based on the results from the orientation recovery, 209 out of 54 405 selected patterns were identified as normal incidence cases, *i.e.* the beam was perpendicular to a cube surface. By studying the speckle spacing, the particle size distribution was found to be in the range 45–61 nm, with a mean size of 52 nm (Fig. 6 ▸). The distribution is consistent with that obtained from STEM measurements (see Fig. 1 ▸), validating the performance of the proposed algorithm. It is worthwhile pointing out that the angular profile is essential for the orientation and size analysis of a large range of size variations.

## 4. Discussion   

**Figure 7:**
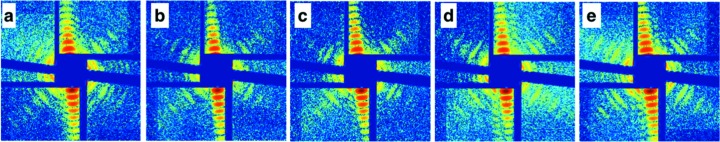
Scattering patterns from particles at the same orientation. The dominant signals (vertical series of speckles) are from the shell and the weaker signals are due to the presence of the Au core. The particles have very similar sizes, indicated by the *q* spacing between the speckles.

The pixel-wise pattern comparison can be useful when the particle size variation is small, as shown in Fig. 3 ▸(*a*). The analysis of XFEL experimental data using cross correlation at the pixel level showed that the patterns can be clustered. The patterns from particles of the same size at similar orientations can be identified. For example, Fig. 7 ▸ shows five XFEL patterns at the same orientation that are identified using cross correlations. The outcome from such an analysis is limited to particles of the same size, therefore the results can be subject to three-dimensional merging and phasing for the given particle size. Pixel-wise comparison cannot be applied directly when the size variation is significant. To get the statistics of the samples with large size variations, new methods are needed that are less sensitive to the particle sizes. In this work, we have demonstrated that the proposed method is robust in pattern comparison when the particle size has a wide distribution, as in the presented core–shell nanoparticle scattering case. Below, we discuss the problems in three-dimensional merging from the experimental data and phase-retrieval challenges when the information at low frequency is missing.

### 4.1. Merging scattering intensity to three-dimensional reciprocal space   

The heterogeneity of the sample particles introduces challenges for orientation recovery, and subsequently prevents merging the data into a three-dimensional diffraction volume. This sample heterogeneity issue becomes a severe bottleneck for the data-processing pipeline. For synthetic nanoparticles, the heterogeneity is often dominated by size variation, because the synthesis of nanoparticles follows well designed protocols to produce the ideal or expected nanoparticles. Here, we constructed an initial reference model based on the particle design and prior information from other measurements (such as the STEM images). The angular profile approach reduces the information to one-dimensional data that are less dependent on the particle size, yet recovers the orientation information at high accuracy. A size-invariant profile is critical for the method to work, otherwise multiple reference models at various sizes must be constructed for a pixel-level intensity comparison, making it computationally intractable.

Using the algorithm presented here, orientation and size information were recovered from single-particle scattering data. We tried to merge the data into a three-dimensional diffraction volume. Patterns corresponding to sizes between 50 and 54 nm were used for three-dimensional merging. The resulting three-dimensional intensity distribution covers about 89.7% of reciprocal space up to a resolution of 7.06 nm. To assess the convergence of the data, we calculated *R*_split_ by randomly dividing the data set into two subsets. The *R*_split_ value was about 0.15 for the resolution range which was well sampled, indicating self-consistency of the data (see Fig. S2 in the supporting information).

**Figure 8:**
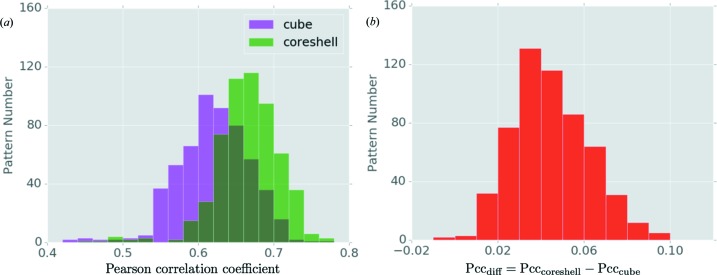
Comparisons between the experimental patterns and two sets of theoretical models. (*a*) The distributions of Pearson correlation coefficients between the experimental and reference models. The green bars show the results with the core–shell model as reference, and the magenta bars are the results using the cubic model (without an octahedral core). (*b*) Histogram of Pearson correlation coefficient (Pcc) difference between the experimental patterns and the best matched patterns from the core–shell and cube reference models. Only two out of 559 patterns favor the cube model over the core–shell model.

The merged data were also compared with the theoretical values of the constructed reference core–shell model. The calculated *R* factor for the intensity was 0.88, too large to conclude that the experimental data are consistent with the model. On the other hand, this can be improved by refining both the orientations of each pattern and the structure of the nanoparticle. As a control, we compared the merged intensities with the cubic shell models *without* the Au core, and the results indicate that the merged data agree better with the core–shell model than with the cube model. A pixel-wise comparison between the experimental pattern and the reference pattern (with corrected size and orientation) for both the cubic model and the core–shell model was carried out for 559 selected scattering patterns. The correlation coefficients indicated that 557 of these patterns are more similar to the core–shell model than to the cubic model (Fig. 8 ▸).

### 4.2. Phase retrieval with information loss at low resolution   

**Figure 9:**
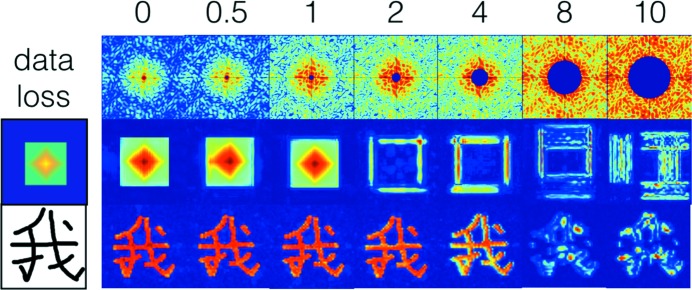
Model reconstructions for two test cases (dense object and sparse object). The numbers on the top row indicate the information loss levels, measured by the number of Shannon pixels missing from the central mask (blue disk). The middle and bottom rows are the reconstruction results for the dense object and sparse object, respectively, with the original objects shown in the first column.

In the data set collected for this study, the intensity at low resolution is missing due mainly to the gaps in the assembled detector, making it difficult to reconstruct real-space models. Using simulated data with missing data at low resolution, we investigated the feasibility of model reconstruction at several levels of information loss (the intensity in the central hole was masked out). The simulated data were generated at the over-sampling ratio of 10. The HIO phase algorithm implemented in *Hawk* was used as the image reconstruction engine (Maia *et al.*, 2010 ▸). Random phases were used for the first iteration, and the feedback factor, β, was set to 0.8 in the phase retrieval experiments. The fraction of support area was reduced from 1 to 0.012 for a dense object and to 0.005 for a sparse object within 5000 iterations, using the shrink-wrap approach (Marchesini *et al.*, 2003 ▸). The phase retrieval results indicate that the information loss within one Shannon pixel can be tolerated by the algorithm using the above protocol. When the information loss at low resolution goes beyond one Shannon pixel, the reconstructed object becomes less accurate, from the boundary of the object to an entirely wrong model. For sparse objects, phase retrieval can be achieved at more severe information loss of up to four Shannon pixels (see Fig. 9 ▸ for a comparison).

In the experimental data set from the LCLS nanoparticle scattering experiments, the information loss is nearly four Shannon pixels. According to our simulation results, two-dimensional reconstructions can barely be achieved using the existing phasing algorithms for this experimental data set, so new algorithms are needed to phase such data. For instance, additional information may be helpful to provide stronger constraints for the model reconstructions.

### 4.3. Statistical characterization of nanoparticles   

Although a three-dimensional model reconstruction was not achieved due to the large amount of information lost at low resolution, sample size distributions were extracted and agree with data measured using the STEM imaging method. This highlights the capability of XFEL single-particle scattering methods for characterizing the properties of nanoparticles. Given that heterogeneity is often an intrinsic characteristic of synthetic nanoparticles such as catalysts, and is often related to function, a statistical analysis becomes necessary to assess sample quality. Using high repetition rate XFELs, with improved sample delivery methods, this single-particle scattering approach can be used for high-throughput studies to measure sample properties at the individual particle level and to quantify the associated heterogeneity.

## 5. Conclusion   

Au–Pd core–shell nanoparticles were intercepted by femtosecond XFEL pulses at LCLS and their scattering signals were detected. These core–shell nanoparticle scattering patterns have useful features to facilitate data analysis. Preliminary results indicate that the scattering signals from individual particles extend to a resolution of about 7.06 nm for Au–Pd core–shell nanoparticles. Computational methods have been developed to characterize the nanoparticles, based on analysis of the scattering patterns. A new method based on an intensity angular profile is described for orientation determination, while features in the shape transforms provide particle size information. The orientation distribution indicates that medium tilting-angle orientations are less populated, reflecting a potential deficiency of the data-analysis algorithm. The size distribution determined from X-ray scattering data is highly consistent with that obtained from electron microscope images, validating the proposed methods for analyzing the scattering patterns of core–shell particles. The merged three-dimensional intensities are self-consistent, and the discrepancy compared with the theoretical values needs to be resolved by refining the pattern orientations and further treatments of the particle heterogeneity. The study also shows that XFEL single-particle scattering can be applied to quantify the statistical properties of nanoparticles at the individual level.

## Supplementary Material

Additional figures. DOI: 10.1107/S2052252517012398/it5013sup1.pdf

## Appendix A

The structure factor can be expressed in the form of a Fourier transform

Let us assume that the particle internal structures are the same, and the size variations can be modeled as a scaling factor *R*, then **r** = , where  spans the unit sphere. The effective form factor *f*(*r*) that describes the particle structure can be treated as a constant ρ within the domain defined by the vector **r** = . Then a model of radius *R* will yield structure factors expressed in the form of

according to the scaling property of the Fourier transform, where *R*_0_ is the size of the reference model. The intensity, the norm of the structure factor, follows a similar relationship. If the integration of the variable *q* is carried out in the domain [0, ∞], the resulting quantity does not depend on the size of the particle, except that the overall intensity will be scaled by . In the cases presented here, the integration cannot be carried out from 0 to infinity due to the constraints of the experimental setup, so instead the integration is limited to the finite domain [*q*_min_, *q*_max_]. Nonetheless, fluctuations in the integrated values due to particle size variation are much smaller than the fluctuations in the raw data recorded at any particular pixel. This is verified from computer simulations, as described in the main text.

## References

[bb1] Aquila, A. *et al.* (2015). *Struct. Dyn.* **2**, 041701. 10.1063/1.4918726 PMC4711616 26798801

[bb2] Barty, A., Kirian, R. A., Maia, F. R. N. C., Hantke, M., Yoon, C. H., White, T. A. & Chapman, H. (2014). *J. Appl. Cryst.* **47**, 1118–1131. 10.1107/S1600576714007626 PMC4038800 24904246

[bb3] Bortel, G. & Tegze, M. (2011). *Acta Cryst.* A**67**, 533–543. 10.1107/S0108767311036269 22011469

[bb4] Bostedt, C., Boutet, S., Fritz, D. M., Huang, Z., Lee, H. J., Lemke, H. T., Robert, A., Schlotter, W. F., Turner, J. J. & Williams, G. J. (2016). *Rev. Mod. Phys.* **88**, 015007.

[bb5] Bostedt, C. *et al.* (2013). *J. Phys. B At. Mol. Opt. Phys.* **46**, 164003.

[bb6] Chapman, H. N. *et al.* (2006). *Nat. Phys.* **2**, 839–843.

[bb7] Dashti, A., Schwander, P., Langlois, R., Fung, R., Li, W., Hosseinizadeh, A., Liao, H. Y., Pallesen, J., Sharma, G., Stupina, V. A., Simon, A. E., Dinman, J. D., Frank, J. & Ourmazd, A. (2014). *Proc. Natl Acad. Sci. USA*, **111**, 17492–17497. 10.1073/pnas.1419276111 PMC4267381 25422471

[bb8] Ekeberg, T. *et al.* (2015). *Phys. Rev. Lett.* **114**, 098102.

[bb9] Emma, P. *et al.* (2010). *Nat. Photon.* **4**, 641–647.

[bb10] Gallagher-Jones, M., Rodriguez, J. A. & Miao, J. (2016). *Q. Rev. Biophys.* **49**, e20.

[bb11] Hosseinizadeh, A., Mashayekhi, G., Copperman, J., Schwander, P., Dashti, A., Sepehr, R., Fung, R., Schmidt, M., Yoon, C. H., Hogue, B. G., Williams, G. J., Aquila, A. & Ourmazd, A. (2017). *Nat. Methods*, **14**, 877–881. 10.1038/nmeth.4395 28805793

[bb12] Hosseinizadeh, A., Schwander, P., Dashti, A., Fung, R., D’Souza, R. M. & Ourmazd, A. (2014). *Philos. Trans. R. Soc. London Ser. B*, **369**, 20130326. 10.1098/rstb.2013.0326 PMC4052863 24914154

[bb13] Kassemeyer, S., Jafarpour, A., Lomb, L., Steinbrener, J., Martin, A. V. & Schlichting, I. (2013). *Phys. Rev. E*, **88**, 042710. 10.1103/PhysRevE.88.042710 24229216

[bb14] Kirian, R. A., Wang, X., Weierstall, U., Schmidt, K. E., Spence, J. C. H., Hunter, M., Fromme, P., White, T., Chapman, H. N. & Holton, J. (2010). *Opt. Express*, **18**, 5713. 10.1364/OE.18.005713 PMC4038330 20389587

[bb15] Li, X. *et al.* (2017). *Sci. Data.* **4**, 170048. 10.1038/sdata.2017.48 PMC5387922 28398334

[bb16] Liang, M. *et al.* (2015). *J. Synchrotron Rad.* **22**, 514–519. 10.1107/S160057751500449X PMC4416669 25931062

[bb17] Liu, H. & Spence, J. C. H. (2016). *Quant. Biol.* **4**, 159–176.

[bb18] Loh, N. D. & Elser, V. (2009). *Phys. Rev. E*, **80**, 026705. 10.1103/PhysRevE.80.026705 19792279

[bb19] Lomb, L., Steinbrener, J., Bari, S., Beisel, D., Berndt, D., Kieser, C., Lukat, M., Neef, N. & Shoeman, R. L. (2012). *J. Appl. Cryst.* **45**, 674–678.

[bb20] Maia, F. R. N. C., Ekeberg, T., van der Spoel, D. & Hajdu, J. (2010). *J. Appl. Cryst.* **43**, 1535–1539.

[bb21] Marchesini, S., He, H., Chapman, H. N., Hau-Riege, S. P., Noy, A., Howells, M. R., Weierstall, U. & Spence, J. C. H. (2003). *Phys. Rev. B*, **68**, 140101.

[bb22] Miao, J., Ishikawa, T., Robinson, I. K. & Murnane, M. M. (2015). *Science*, **348**, 530–535. 10.1126/science.aaa1394 25931551

[bb23] Ourmazd, A., Schwander, P. & Phillips, G. N. Jr (2010). *SPIE Optical Engineering + Applications*, Vol. 7800, *Image Reconstruction from Incomplete Data VI*, edited by P. J. Bones, M. A. Fiddy and R. P. Millane, abstract 780002. Bellingham, Washington, USA: SPIE.

[bb24] Robinson, I. K., Vartanyants, I. A., Williams, G. J., Pfeifer, M. A. & Pitney, J. A. (2001). *Phys. Rev. Lett.* **87**, 195505. 10.1103/PhysRevLett.87.195505 11690423

[bb25] Scheres, S. H. W. (2012). *J. Struct. Biol.* **180**, 519–530. 10.1016/j.jsb.2012.09.006 PMC3690530 23000701

[bb26] Shneerson, V. L., Ourmazd, A. & Saldin, D. K. (2008). *Acta Cryst.* A**64**, 303–315. 10.1107/S0108767307067621 18285625

[bb27] Takahashi, Y., Suzuki, A., Zettsu, N., Oroguchi, T., Takayama, Y., Sekiguchi, Y., Kobayashi, A., Yamamoto, M. & Nakasako, M. (2013). *Nano Lett.* **13**, 6028–6032. 10.1021/nl403247x 24274169

[bb28] Weierstall, U., Spence, J. C. H. & Doak, R. B. (2012). *Rev. Sci. Instrum.* **83**, 035108. 10.1063/1.3693040 22462961

[bb29] Williams, G. J., Pfeifer, M. A., Vartanyants, I. A. & Robinson, I. K. (2003). *Phys. Rev. Lett.* **90**, 175501. 10.1103/PhysRevLett.90.175501 12786079

[bb30] Yang, C.-W., Chanda, K., Lin, P.-H., Wang, Y.-N., Liao, C.-W. & Huang, M. H. (2011). *J. Am. Chem. Soc.* **133**, 19993–20000. 10.1021/ja209121x 22091631

