# Supplementary material for: Merging single-shot XFEL diffraction data from inorganic nanoparticles: a new approach to size and orientation determination
Source: IUCrJ. 2017 Sep 22;4(Pt 6):741–50. doi: 10.1107/S2052252517012398 (PMC5668859; doi:10.1107/S2052252517012398)
Supplement: Supplementary file 1 [file m-04-00741-sup1.pdf]

IUCrJ

Volume 4 (2017)

Supporting information for article:

Merging single-shot XFEL diffraction data from inorganic nanoparticles: a new approach to size and orientation determination

Xuanxuan Li, Stephan Kassemeyer, John C. H. Spence, Ilme Schlichting, Brenda G. Hogue and Haiguang Liu

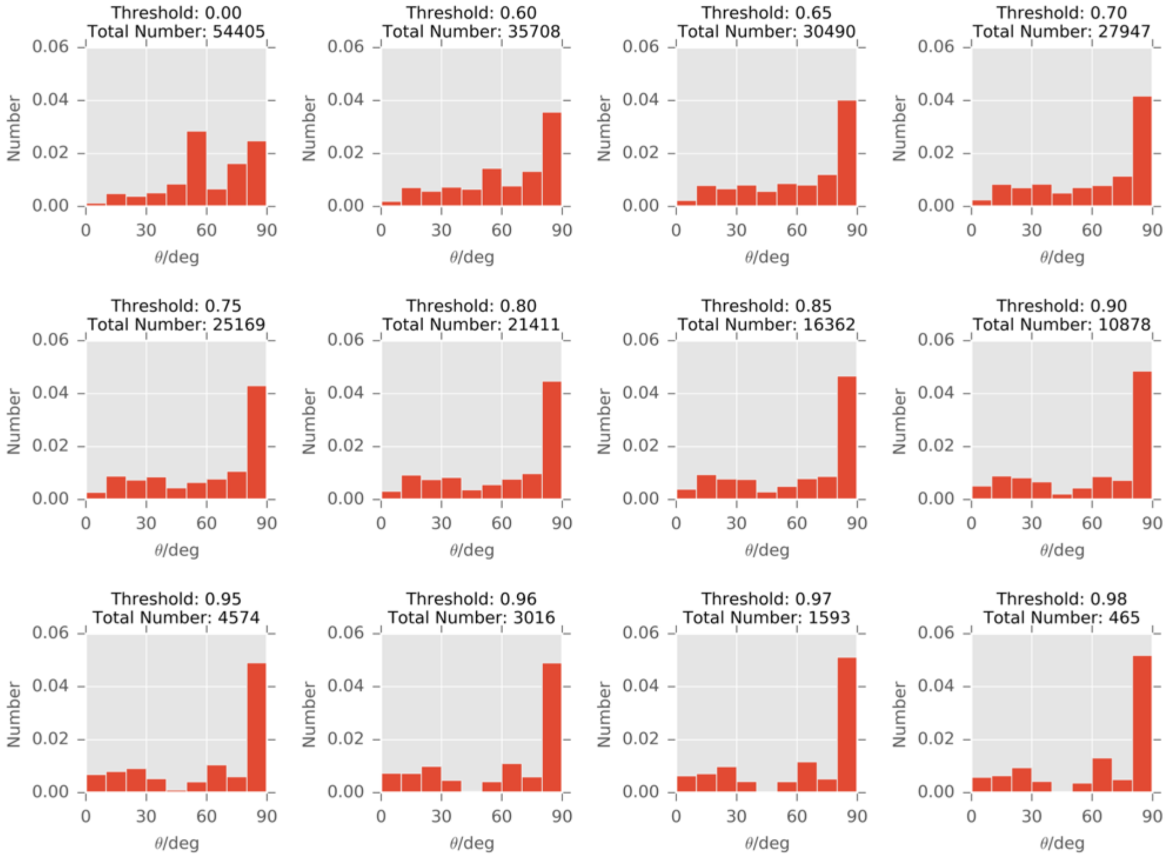

Fig. S1. **Tilting angle distributions at different thresholds.** If we increase the correlation coefficient threshold from 0 to 0.98, the number of patterns decreases rapidly and confidence level increases. The distribution becomes convergent when the threshold is increased to 0.80.

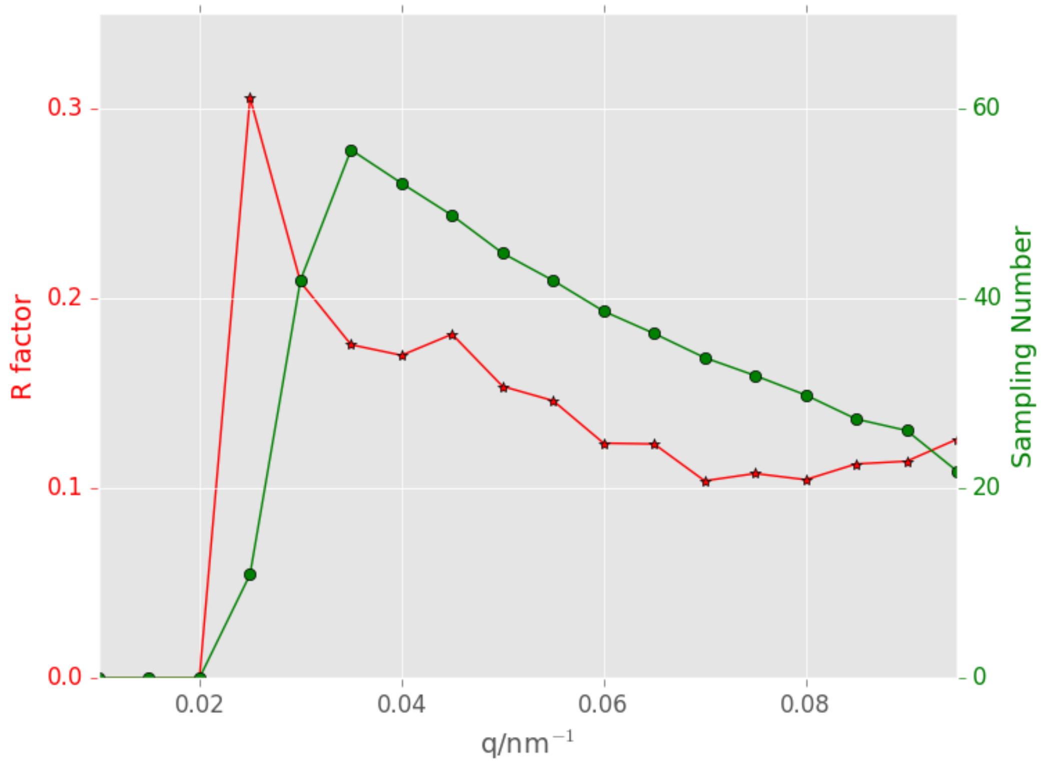

Fig S2. **The R-factors between merged intensities from subsets of data.** The red curve shows the R-split factors at resolution shells indicated by the q values. The sampling number (green curve) indicates the average number of valid measurements at each resolution shell in reciprocal space.
